# Equity by design: integrating a Deprivation Index into digital platforms for breast cancer and chronic disease prevention—lessons from the ELISAH project

**DOI:** 10.3389/fpubh.2025.1682320

**Published:** 2025-10-30

**Authors:** Silvia Ussai, Arantza Sanvisens Bergé, Ellas Spyratou, Patrizia Pasanisi, Roberto Boffi, Mykhailo Mosora, Giovanna Tagliabue, Alessandro Borgini, Paolo Contiero

**Affiliations:** ^1^IRCCS Istituto Nazionale dei Tumori di Milano, Milan, Italy; ^2^Catalan Institute of Oncology (ICO), Barcelona, Spain; ^3^National and Kapodistrian University of Athens, Athens, Greece; ^4^Carpathian Institute of Analytics “FrankoLytics”, Kyiv, Ukraine

**Keywords:** equity, conflict-affected settings, cancer prevention, digital health, ELISAH

## Abstract

**Background:**

Socio-economic inequalities are a major determinant of chronic disease risk and access to preventive services. Deprivation indices can quantify these disparities, but very few studies have considered them when examining the relationship between breast cancer incidence, prognosis, and particulate matter as a potential effect modifier. Moreover, these indices have rarely been used as active tools in service delivery. Digital health technologies offer an opportunity to embed equity into chronic disease prevention through the real-time integration of socio-economic metrics. The ELISAH project developed a digital prevention framework that incorporates a Deprivation Index to support strategies targeting breast cancer and other chronic diseases.

**Methods:**

A descriptive, policy-oriented analysis was conducted using the ELISAH framework as a case study. Nationally validated Deprivation Indices were embedded into the ELISAH platform, which integrates socio-economic, demographic, environmental, and behavioral data. A conflict-sensitive version was developed to address the needs of internally displaced populations and service disruptions in fragile health systems. The Index was configured to support both population-level equity mapping and individual-level tailoring of preventive interventions.

**Results:**

The Deprivation Index has been technically integrated into the digital platform and piloted in both urban and conflict-affected settings. Early implementation confirmed the feasibility of harmonizing socio-economic indicators with digital engagement data and environmental exposures. The platform’s architecture enables dynamic monitoring of inequalities and the implementation of adaptive prevention strategies. While comprehensive outcome data are pending full-scale deployment, pilot mapping has established proof-of-concept for embedding equity into digital health systems by design.

**Discussion:**

Embedding a Deprivation Index within a digital prevention platform demonstrates how equity can be incorporated into both policy-oriented observational studies and as an active design principle. This dual application supports personalized interventions and real-time, equity-driven resource allocation. The ELISAH approach highlights the potential of digital health tools to address socio-economic and conflict-related disparities in chronic disease prevention.

**Conclusion:**

Integrating a Deprivation Index into digital prevention systems offers a scalable model for addressing health inequalities in chronic disease management. The ELISAH framework demonstrates the feasibility of making equity a foundational element of digital platforms, enabling personalized prevention, informed policy planning, and improved resilience in both stable and crisis-affected health systems.

## 1 Introduction

Socio-economic inequalities are among the strongest predictors of chronic disease burden in Europe, influencing both incidence and outcomes for conditions such as breast cancer. Multiple studies have demonstrated that women living in socio-economically deprived areas are more likely to be diagnosed at later stages and to have lower participation rates in preventive programs, including organized screening initiatives (1, 2). To measure and monitor these disparities, deprivation indices have been widely used in spatial epidemiology. In parallel, digital health technologies—including mobile applications, real-time registries, and integrated data platforms—are increasingly promoted as essential tools to support personalized prevention and scale interventions for non-communicable diseases (NCDs) (3, 4). Despite their potential, systematic reviews have highlighted that equity considerations are often insufficiently embedded in digital health design, resulting in interventions that risk widening, rather than reducing, existing disparities (5).

The ELISAH project (European Linkage of Initiative from Science to Action in Health), aiming to reduce the burden of breast cancer by acting on modifiable risk factors and funded under the EU4Health programme, explicitly addresses this gap by embedding a measure of socio-economic inequality, the Deprivation Index, into its digital ecosystem. ELISAH acronym is “European Linkage from Science to Actions,” the project was designed to translate scientific knowledge and observations made by ad-hoc studies into actions. To be sure that the “translation” could take into account socio-economic inequalities in a scientifically sounded way we considered mandatory to use the same inequality metrics throughout the different phases of the study and linking the observational analysis of ELISAH to quantify the distribution of breast cancer risk factor on populations and intervention studies of ELISAH aiming to remove or mitigate the same risk factors. Socio-economic inequalities are taking into account for all the risk factors on which ELISAH is working: lifestyle changes in diet, physical activities and smoking habits, urban assets in relation to breast cancer risk, atmospheric pollution.

Considering that women participation to intervention for lifestyle changes may be biased by upper-class women selection, analysis of the urban assets in relation to breast cancer risk in some European cities are also evaluated according to the neighborhood socio-economic status, atmospheric pollution in the same way analyzed in relation to area socio-economic status.

The use of a common Deprivation Index enhances the generalizability of the ELISAH results. As an example, the collection of information on BMI, performed by the observational study on population-based Cancer Registries, and its impact on breast cancer survival could be generalized to other population showing a different Deprivation profile thanks to the population classification into different Deprivation scores.

ELISAH combines geocoded socio-economic and environmental data with cancer registry information and behavioral interventions delivered via a mobile platform, aiming to integrate equity into prevention by design. A Ukraine-specific adaptation of the Deprivation Index was developed to account for conflict-related displacement, while urban pilots linked the Index to environmental exposure data to capture intersectional determinants of chronic disease risk (6).

By linking deprivation metrics with digital behavioral tools and population registries, ELISAH provides a live example of how equity frameworks can be operationalized in digital chronic disease prevention. This article uses ELISAH as a case study to examine how deprivation indices can be integrated into digital health platforms to guide policy, target interventions, and support the reduction of socio-economic disparities in chronic disease prevention and management.

## 2 Methods

The first choice when working with socio-economic inequalities regards the kind of measure. We choose to use an area-based inequality measure, the Deprivation Index, instead of an individual-based indicator because of the ELISAH aim to evaluate “environmental” condition more deeply than individual ones. Many Deprivation Indexes are described in literature (12). The European Deprivation Index (EDI), developed to harmonize socio-economic measurement across EU Member States, was not available in Greece and Ukraine forcing us to use national Deprivation Indexes. Nationally developed Deprivation Indexes was used in breast cancer studies in Italy and in Spain, in the Attica region of Greece a Deprivation Index was developed, tested and applied to local urban analysis. These applications reassured us about the reliability of these metrics. In Ukraine we piloted the set-up of a specific Ukraine Deprivation Index incorporating displacement and conflict-related variables to address the specific vulnerabilities of internally displaced persons (IDPs) (7).

**Figure 1:**
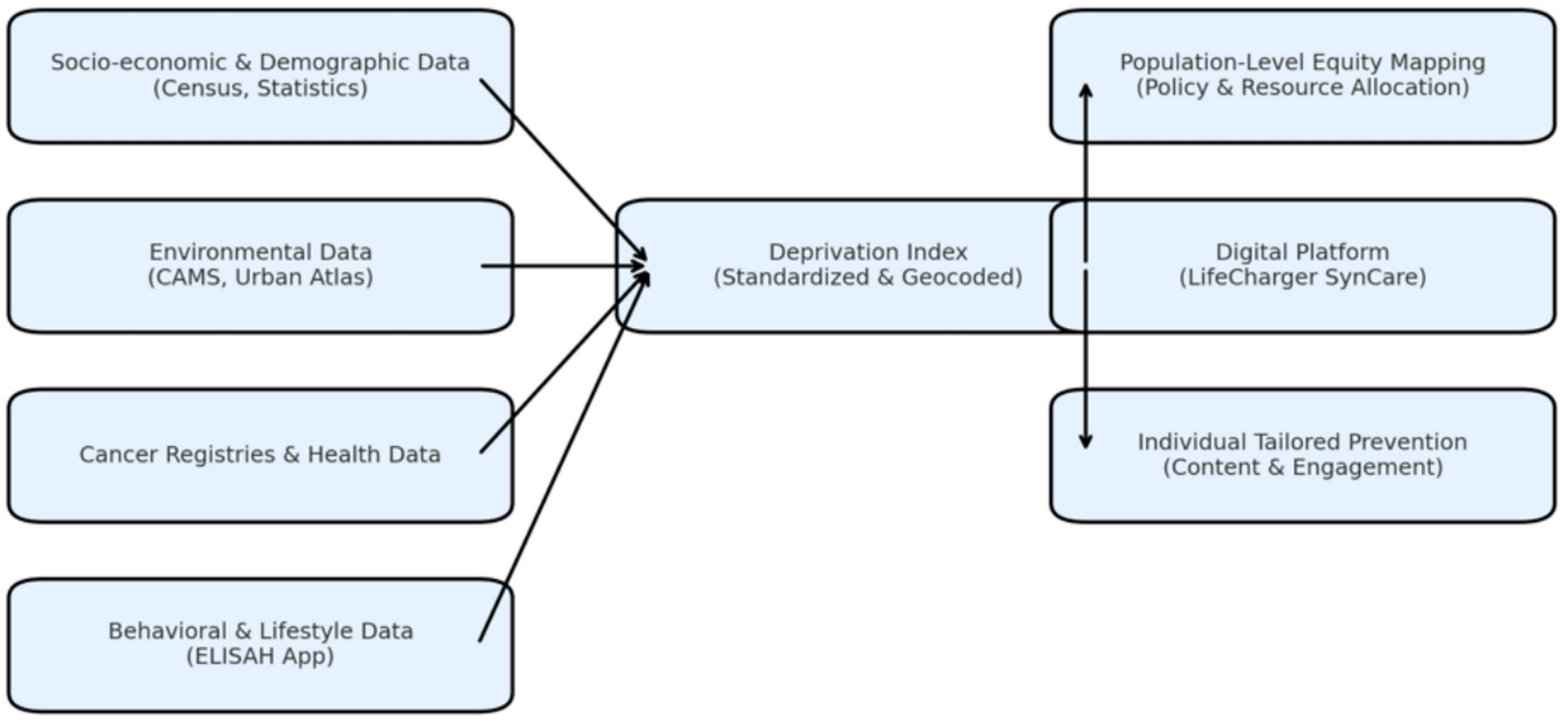
Conceptual architecture for integrating a Deprivation Index into digital chronic disease prevention platforms.

The design allows for two functions: (1) population-level equity mapping using geocoded deprivation scores to quantify the distribution of risk factors in the population under analysis, and (2) individual-level tailoring of prevention content and engagement strategies based on socio-economic profile. This creates a dynamic feedback loop between digital service delivery and equity monitoring (Figure 1).

The planned data architecture links the Deprivation Index to multiple sources: population based cancer registries, behavioral and lifestyle data collected via the ELISAH App, and environmental datasets including air pollution (Copernicus) and green space mapping (Urban Atlas) and land use (CORINE Land Cover). Although full-scale linkage is pending ongoing data collection, a pilot mapping of the Index structure onto available registry and population data has been completed to validate feasibility.

Given the absence of finalized outcome data, the present analysis does not include statistical results. Instead, it provides a conceptual and implementation-level description of the methodology, digital integration, and expected policy applications of the Deprivation Index. The focus is on demonstrating how this equity-driven design can be embedded into digital prevention systems to support both individual risk stratification and population-level resource allocation in line with EU and WHO priorities for reducing health inequalities (8).

## 3 Results

### 3.1 A conceptualization and pilot development of the Deprivation Index

Within the ELISAH framework, the Deprivation Index was designed as a digital equity layer capable of integrating socio-economic, demographic, and environmental indicators into a single geocoded measure. The methodological blueprint validated national Deprivation Index to national contexts, with a Ukraine-specific variant incorporating displacement and conflict-related variables to capture the needs of internally displaced persons (9). At this stage, the Index exists as a harmonized framework with pilot mapping exercises completed in selected regions to validate feasibility and linkage with population and registry data.

The Deprivation Index has then been embedded within the ELISAH digital app-dashboard ecosystem, connecting the patient-facing ELISAH App, clinical dashboard, and back-end analytics. The integration is configured to support two key outputs: population-level equity mapping to inform policy and resource allocation, and individual-level risk stratification to adapt prevention content based on socio-economic context. Initial technical validation confirmed seamless interoperability between the Index architecture and the platform’s data analytics module (8).

### 3.2 B application in conflict zones

In Ukraine, the adapted Index has been aligned with local census and health system data structures, with the additional layer of IDP-related variables tested in a pilot linkage. Early mapping exercises demonstrated that the Index can identify areas of heightened vulnerability and inform digital intervention targeting. Urban pilot regions in other partner countries have used the Index to overlay socio-economic deprivation with environmental exposures such as NO₂ concentrations and green space coverage, establishing a baseline for future population-level equity analyses (10).

### 3.3 C anticipated outputs and policy relevance

Although comprehensive outcome data are not yet available, the architecture is designed to produce live inequality metrics once fully linked with cancer registries and digital engagement data. This will allow health authorities to visualize equity gaps and adapt prevention strategies accordingly. The proof-of-concept developed under ELISAH positions the Deprivation Index as a scalable tool that can be integrated into national and cross-border digital health infrastructures to embed equity monitoring directly into chronic disease prevention systems (5).

## 4 Discussion

Integrating a Deprivation Index into digital platforms for chronic disease prevention offers a way to embed equity into the core of health system transformation. Traditionally, deprivation indices have been retrospective tools for mapping socio-economic gradients in disease burden (9) but their application in specific setting such as the link between breast cancer and particulate matter exposure are very scarce and recommendations were published encouraging similar analysis (2). The approach piloted through ELISAH reframes them as dynamic, operational components of prevention programs, capable of guiding both individual-level interventions and population-level resource allocation in real time.

A key innovation of this model is its adaptability to different health system environments, including conflict-affected zones. In such contexts, inequalities are amplified by population displacement, disrupted service infrastructure, and reduced access to preventive care. By incorporating conflict-specific indicators such as IDP density and service disruption, the Deprivation Index provides a structured way to identify and prioritize vulnerable groups even when traditional health information systems are compromised (11).

Embedding this equity layer within a digital platform allows targeted interventions to reach populations who are typically invisible to routine prevention strategies in humanitarian and post-conflict settings. The ELISAH pilot illustrates that digital health tools can bridge gaps between epidemiological evidence and operational response when designed with conflict-sensitive equity metrics.

At a policy level, integrating deprivation-based metrics into digital chronic disease platforms aligns with Europe’s Beating Cancer Plan, EU4Health priorities, and the WHO Global Strategy on Digital Health. The conflict-zone application adds a critical dimension by demonstrating that the same equity-driven design can inform EU humanitarian health responses and national resilience planning. It highlights the potential of digital tools to serve as both prevention infrastructure and crisis-response mechanisms.

The urban pilots also show the added value of combining socio-economic deprivation with environmental indicators such as air quality and green space. This dual-layer approach identifies compounded vulnerabilities and supports cross-sector interventions that combine digital prevention with urban planning and environmental health policy (10).

The next steps involve full linkage of the Deprivation Index to cancer registry and digital engagement data to generate real-world evidence of its impact. Beyond breast cancer, the methodology offers a framework for equity-focused digital strategies in other chronic diseases and in public health preparedness. In conflict zones, integrating the Index into mobile platforms could support rapid needs assessments, equitable resource allocation, and continuity of prevention programs during service disruptions.

## 5 Conclusions and policy recommendations

The integration of a Deprivation Index into digital prevention platforms represents a strategic opportunity to embed equity into the digital transformation of chronic disease management. Based on the ELISAH framework and early implementation, several key policy directions emerge.

First, national and EU-level digital health strategies should adopt standardized deprivation-based metrics as part of all digital prevention infrastructures. Embedding such indices in platforms aligned with the European Health Data Space would enable cross-country interoperability and support monitoring of health inequalities across Member States (4). Second, conflict-sensitive adaptations of deprivation indices should be developed as part of preparedness and humanitarian health planning. Incorporating displacement and service disruption indicators can ensure that digital platforms maintain equity even in fragile and crisis-affected contexts (7). Third, deprivation metrics should be dynamically linked to cancer registries and behavioral data streams within digital ecosystems to shift from retrospective inequality mapping to real-time monitoring. This would allow health systems to continuously assess and address access gaps as digital interventions scale (8). Fourth, digital prevention tools must integrate equity-informed personalization. Tailoring content and engagement strategies based on socio-economic context can mitigate digital divides and improve reach to vulnerable groups, ensuring that digital innovation reduces rather than amplifies inequalities (5).

Finally, public financing mechanisms should include dedicated support for equity monitoring tools within digital health platforms. Sustainable funding is critical to maintain and update deprivation indices, ensure their integration into routine prevention services, and translate them into actionable policy insights (3).

In conclusion, the ELISAH model demonstrates how a Deprivation Index can be operationalized within a digital chronic disease prevention ecosystem to deliver “Equity by Design.” Although comprehensive data are still forthcoming, the conceptual framework and early implementation show that embedding socio-economic and conflict-sensitive metrics into digital health platforms is both feasible and policy-relevant. This approach aligns with WHO and EU priorities on reducing health inequalities and offers a scalable roadmap for integrating equity into the future of digital chronic disease prevention across both stable and conflict-affected settings.

Socio-economic and demographic indicators (from census and statistical datasets), environmental data (air pollution and green space metrics), cancer registry information, and behavioral data from the ELISAH mobile application are combined to generate a standardized, geocoded Deprivation Index. The Index is embedded within the LifeCharger SynCare digital ecosystem, enabling both population-level equity mapping for policy and resource allocation and individual-level tailoring of prevention content and engagement. This architecture illustrates the operationalization of “Equity by Design” in digital health by linking socio-economic context directly to intervention delivery and monitoring.
